# Reinforcing Increase of *ΔT_c_* in MgB_2_ Smart Meta-Superconductors by Adjusting the Concentration of Inhomogeneous Phases

**DOI:** 10.3390/ma14113066

**Published:** 2021-06-04

**Authors:** Yongbo Li, Guangyu Han, Hongyan Zou, Li Tang, Honggang Chen, Xiaopeng Zhao

**Affiliations:** Smart Materials Laboratory, Department of Applied Physics, Northwestern Polytechnical University, Xi’an 710072, China; 2014100616@mail.nwpu.edu.cn (Y.L.); guangyu@mail.nwpu.edu.cn (G.H.); 2019202522@mail.nwpu.edu.cn (H.Z.); 2018202416@mail.nwpu.edu.cn (L.T.); 2017100698@mail.nwpu.edu.cn (H.C.)

**Keywords:** MgB_2_, EL inhomogeneous phase, inject energy, SMSCs, *ΔT_c_*

## Abstract

Incorporating with inhomogeneous phases with high electroluminescence (EL) intensity to prepare smart meta-superconductors (SMSCs) is an effective method for increasing the superconducting transition temperature (*T_c_*) and has been confirmed in both MgB_2_ and Bi(Pb)SrCaCuO systems. However, the increase of *ΔT_c_* (*ΔT_c_* = *T_c_* ‒ *T_cpure_*) has been quite small because of the low optimal concentrations of inhomogeneous phases. In this work, three kinds of MgB_2_ raw materials, namely, ^a^MgB_2_, ^b^MgB_2_, and ^c^MgB_2_, were prepared with particle sizes decreasing in order. Inhomogeneous phases, Y_2_O_3_:Eu^3+^ and Y_2_O_3_:Eu^3+^/Ag, were also prepared and doped into MgB_2_ to study the influence of doping concentration on the *ΔT_c_* of MgB_2_ with different particle sizes. Results show that reducing the MgB_2_ particle size increases the optimal doping concentration of inhomogeneous phases, thereby increasing *ΔT_c_*. The optimal doping concentrations for ^a^MgB_2_, ^b^MgB_2_, and ^c^MgB_2_ are 0.5%, 0.8%, and 1.2%, respectively. The corresponding *ΔT_c_* values are 0.4, 0.9, and 1.2 K, respectively. This work open a new approach to reinforcing increase of *ΔT_c_* in MgB_2_ SMSCs.

## 1. Introduction

According to BCS theory, McMillan theoretically calculated the upper limit of the critical temperature (*T_c_*) of conventional BCS superconductors to be 40 K, which is called the McMillan limit temperature [1,2]. Although the *T_c_* of conventional superconductors has an upper limit, the search for high-*T_c_* superconducting materials has been continuous. High-temperature superconductors [3,4], iron-based superconductors [5,6], high-pressure superconductors [7,8,9,10], and photo-induced superconductors [11,12] have been gradually studied and discovered. However, these new superconducting materials are not simple conventional superconductors. Breaking the McMillan limit temperature remains a challenge for conventional BCS superconductors. In 2001, the superconductivity of MgB_2_ was discovered [13]. The excellent superconductivity, simple preparation process, and especially high *T_c_* of MgB_2_ quickly aroused great interest in the scientific community and led scholars to believe that the McMillan limit temperature may finally be surpassed [14,15,16,17,18,19]. Various methods have been applied to improve the superconductivity of MgB_2_ [20,21,22,23,24], which would not only improve the practical application of MgB_2_ but also help transcend the McMillan limit temperature and further elucidate the superconducting mechanism. Chemical doping is often used to study superconductivity. Unfortunately, many experimental results confirm that this method reduces the *T_c_* of MgB_2_ [25,26,27,28,29,30]. Thus far, no useful strategy for improving the *T_c_* of MgB_2_ is yet available.

Metamaterial mainly refers to materials made up of two or more media, which can produce new properties that are not found in a single medium. Meta-method is often used to achieve some special properties and provides new ways of improving the *T_c_* of materials [31,32,33]. In 2007, our group proposed a method based on the structural design of metamaterials for increasing the *T_c_* of superconductors [34,35]. In this method, electroluminescence (EL) materials are directly doped into a superconductor to form a smart meta-superconductor (SMSC). The external field added during the measurement of the *T_c_* of SMSC with a four-probe method can excite the inhomogeneous phases to generate EL, achieving the purpose of strengthening the Cooper pairs, resulting the change of *T_c_* in macroscopic. A SMSC is a material whose *T_c_* can be adjusted and improved by the stimulus of external field, which is a new property and cannot be achieved by traditional doping with a second phase [36,37,38,39,40,41,42]. Our group subsequently conducted a series of studies, mainly using MgB_2_ as the base superconducting material and Y_2_O_3_:Eu^3+^ as the base EL material [36,37,38]. The results obtained in these studies show that unlike conventional chemical doping, which consistently reduces the *T_c_* of MgB_2_, the SMSC method of doping EL materials could help increase the *T_c_* of MgB_2_. The same conclusions were drawn from substituting the inhomogeneous phase with YVO_4_:Eu^3+^ or luminescent nanocomposite Y_2_O_3_:Eu^3+^/Ag [39,40] and replacing MgB_2_ with Bi(Pb)SrCaCuO [41,42]. The effectiveness of improving the *T_c_* of superconducting materials through the SMSC method by doping with EL inhomogeneous phases has been proven, but the *ΔT_c_* (*ΔT_c_* = *T_c_* − *T_cpure_*) values obtained are generally small (0.2–0.4 K). Our previous results show that the SMSC method can only improve *T_c_* at low concentrations of inhomogeneous phases and leads to a small *ΔT_c_*, greatly hindering the further improvement of the *T_c_* of MgB_2_. Very recently, our group has increased the *T_c_* of smart meta-superconductor Bi(Pb)SrCaCuO by adjusting the content of inhomogeneous phase [42], implying that the *T_c_* of MgB_2_ SMSC can be further improved through the similar method.

In this work, three types of MgB_2_ raw materials, namely, ^a^MgB_2_, ^b^MgB_2_, and ^c^MgB_2_, were prepared with particle sizes decreasing in order. Two types of inhomogeneous phases, namely, Y_2_O_3_:Eu^3+^ and Y_2_O_3_:Eu^3+^/Ag, were also prepared based on our previous preparation method [43,44]. Two other types of non-EL dopants, namely, Y_2_O_3_ and Y_2_O_3_:Sm^3+^, were also prepared for comparison. These four types of dopants were incorporated into MgB_2_, and the change of *T_c_* was studied. The results show that the *T_c_* of MgB_2_ doped with non-EL Y_2_O_3_ and Y_2_O_3_:Sm^3+^ was lower than that of pure MgB_2_ (*ΔT_c_* < 0). By contrast, EL inhomogeneous phases Y_2_O_3_:Eu^3+^ and Y_2_O_3_:Eu^3+^/Ag increased the *T_c_* (*ΔT_c_* > 0), and the optimal doping concentration of the inhomogeneous phases increased from 0.5% to 1.2% with the decrease of MgB_2′_s particle size. The optimal doping concentrations for ^a^MgB_2_, ^b^MgB_2_, and ^c^MgB_2_ were 0.5%, 0.8%, and 1.2%, respectively. The corresponding *ΔT_cs_* were 0.4 K, 0.9 K, and 1.2 K, which exhibit significant improvements compared with the *ΔT_cs_* (0.2–0.4 K) in previous work [36,37,38,39,40]. Such an improvement of *T_c_* is a novel property given that all the experiments before our work confirmed that doping a second phase decreased the *T_c_* of MgB_2_.

## 2. Model

**Figure 1:**
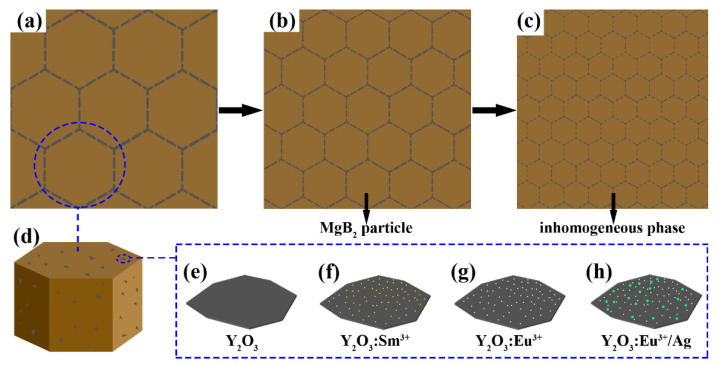
The models of MgB_2_ SMSCs prepared using (**a**) ^a^MgB_2_ (Φ_a_ < 30 μm), (**b**) ^b^MgB_2_ (Φ_b_ < 15 μm), and (**c**) ^c^MgB_2_ (Φ_c_ < 5 μm) as raw materials. Schematic depictions of (**d**) a particle of MgB_2_ SMSC, (**e**) Y_2_O_3_, (**f**) Y_2_O_3_:Sm^3+^, (**g**) Y_2_O_3_:Eu^3+^, and (**h**) Y_2_O_3_:Eu^3+^/Ag. The morphology of Y_2_O_3_, Y_2_O_3_:Sm^3+^, Y_2_O_3_:Eu^3+^, and Y_2_O_3_:Eu^3+^/Ag is flaky with surface size of approximately 20 nm and thickness of approximately 2.5 nm [40].

Figure 1a–c show the cross-sectional view of MgB_2_ SMSCs models prepared using ^a^MgB_2_ (Φ_a_ < 30 μm), ^b^MgB_2_ (Φ_b_ < 15 μm), and ^c^MgB_2_ (Φ_c_ < 5 μm) as raw materials. Φ_a_, Φ_b_, and Φ_c_ refer to the particle sizes of ^a^MgB_2_, ^b^MgB_2_, and ^c^MgB_2_ powders, which will be described in detail at the experiment section. The brown hexagons represent the MgB_2_ particles, and the gray dashed lines represent the flakes of inhomogeneous phase with the surface size of approximately 20 nm and thickness of approximately 2.5 nm [40,45]. The flakes of Y_2_O_3_, Y_2_O_3_:Sm^3+^, Y_2_O_3_:Eu^3+^, and Y_2_O_3_:Eu^3+^/Ag mainly gather on the surfaces of the MgB_2_ particles as shown in Figure 1d. Figure 1e–h present the schematics of Y_2_O_3_, Y_2_O_3_:Sm^3+^, Y_2_O_3_:Eu^3+^, and Y_2_O_3_:Eu^3+^/Ag, respectively. The gray flake represents Y_2_O_3_. The yellow, white, and green points represent Sm, Eu, and Ag. Obviously, the introduction of these four dopants inevitably reduces the *T_c_* of MgB_2_. This is mainly because the dopants are not superconductors, which is unfavorable for the superconductivity of MgB_2_, like the impurity phase of MgO in MgB_2_. For convenience, the reduction in *T_c_* caused by introducing the dopants is referred to as the impurity effect [36,37,38,39,40,41,42]. Non-EL dopants Y_2_O_3_ and Y_2_O_3_:Sm^3+^ can only decrease *T_c_* for the introduction of the impurity effect. Unlike Y_2_O_3_ and Y_2_O_3_:Sm^3+^, introducing EL Y_2_O_3_:Eu^3+^ and Y_2_O_3_:Eu^3+^/Ag may increase the *T_c_*, which is referred to as the EL exciting effect [36,37,38,39,40,41,42]. Incorporating with inhomogeneous phases has already been confirmed to be an effective method of increasing the *T_c_* for both MgB_2_ and Bi(Pb)SrCaCuO systems. The variation of *T_c_* is often associated with the change of electron density. However, in the experiments, the inhomogeneous phases do not react with MgB_2_ and the diffusion between the inhomogeneous phases and MgB_2_ particles is difficult under the current preparation process and conditions. As a result, the dopants only exist between the MgB_2_ particles as shown in Figure 1a–c and cannot change the electron density significantly. Therefore, in principle, the electron density is not the key tuning parameter for the variation of *T_c_*. Although the mechanism for this method remains unclear, we intend to interpret this phenomenon in terms of EL of inhomogeneous phases based on the results of our experiments. During the measurements, the applied external electric field forms local electric fields in the superconductor, which could excite the inhomogeneous phase to produce EL. The generated EL excites the electrons to inject energy, which is favorable to strengthen the Cooper pairs and enables the increase in *T_c_*. However, the completeness of this interpretation needs further demonstration given that the photons may disrupt Cooper pairs. Anyway, further study is required to build a relatively complete theory, especially for such a new experimental phenomenon.

A distinct competition exists between the impurity effect and EL exciting effect. *T_c_* would be improved (*ΔT_c_* > 0) when EL exciting effect dominates; otherwise, introducing the inhomogeneous phase would decrease *T_c_* (*ΔT_c_* < 0). During the preparation process, the impurity effect should be reduced as extensively as possible, and the EL exciting effect should be enhanced to obtain samples with a high *T_c_*. The resulting superconductor is called a SMSC, and the *T_c_* of which can be improved and adjusted by incorporating EL inhomogeneous phases [36,37,38,39,40,41,42], which is a new property and cannot be achieved by traditional doping with a second phase. However, the *ΔT_cs_* obtained in our previous work through the SMSC method are quite small. The low doping concentrations of inhomogeneous phases greatly hindered the further improvement of *T_c_*. To further improve the *ΔT_c_* of MgB_2_, the doping concentration of the inhomogeneous phase must be increased to enhance the EL exciting effect. However, the impurity effect inevitably increases with the increasing doping concentration, as analyzed above. The results of our previous work show that the impurity effect tends to dominate at high concentrations, which is not conducive to the *T_c_* of the sample. This phenomenon is principally caused by the agglomeration of excessive inhomogeneous phase flakes, which cannot disperse well in the sample to improve *T_c_* at concentrations exceeding the optimal value. A simple strategy to solve this problem is to reduce the particle size of MgB_2_ as shown in Figure 1a–c. It can be seen that reducing the particle size would increase the optimal doping concentration of the inhomogeneous phase. The inhomogeneous phase flakes can disperse well in the sample with small particle size and fully exert the EL exciting effect to further increase *ΔT_c_*. Such a strategy has already been successfully applied to increase the *T_c_* of smart meta-superconductor Bi(Pb)SrCaCuO [42].

## 3. Experiment

Y_2_O_3_, Y_2_O_3_:Sm^3+^, Y_2_O_3_:Eu^3+^, and Y_2_O_3_:Eu^3+^/Ag were prepared by a hydrothermal method [40,44]. Briefly, a certain amount of Y_2_O_3_ and Eu_2_O_3_ were weighed and dissolved in HCl to make a precursor. The precursor was dissolved in benzyl alcohol and stirred with a magnetic stirrer. A certain amount of octylamine and AgNO_3_ was added dropwise into the beaker in turn. Then the mixture was transferred to a high-pressure reaction kettle, which was then placed in a drying oven and kept at 250 °C for 24 h. Thereafter, the reaction kettle was naturally cooled to room temperature. The precipitate was washed several times with absolute ethanol to remove impurities and then separated from the solution by centrifugation, precipitation, and drying. The obtained solids were placed in a high-temperature tube furnace and heated at 800 °C for 24 h to form a white powder. After illumination, Y_2_O_3_:Eu^3+^/Ag was obtained. The same procedure was carried out prepare Y_2_O_3_, Y_2_O_3_:Eu^3+^, and Y_2_O_3_:Sm^3+^ by controlling the addition of Eu_2_O_3_ and AgNO_3_ and replacing Eu_2_O_3_ with Sm_2_O_3_. The morphology of Y_2_O_3_, Y_2_O_3_:Sm^3+^, Y_2_O_3_:Eu^3+^, and Y_2_O_3_:Eu^3+^/Ag is flaky with surface size of approximately 20 nm and thickness of approximately 2.5 nm [40,45].

Three types of MgB_2_ raw materials marked with ^a^MgB_2_, ^b^MgB_2_, and ^c^MgB_2_ were prepared in this work. Φ_a_, Φ_b_, and Φ_c_ refer to the particle sizes of ^a^MgB_2_, ^b^MgB_2_, and ^c^MgB_2_ powders. A 500-mesh sieve was used to sift MgB_2_ powder (99%, 100 mesh, Alfa Aesar) to prepare ^a^MgB_2_, indicating that Φ_a_ < 30 μm. ^b^MgB_2_ was prepared by sifting ^a^MgB_2_ powder through vacuum filtration with a pore size of about 15 μm, indicating that Φ_b_ < 15 μm. Meanwhile, Mg and nano boron powder sifted through vacuum filtration with the pore size of about 5 μm were applied to prepare MgB_2_ powder by the traditional sintering process. The obtained MgB_2_ powder was then sifted through vacuum filtration with the pore size of about 5 μm to prepare ^c^MgB_2_, indicating that Φ_c_ < 5 μm. MgB_2_-based superconductors were synthesized by an ex situ preparation process, which is described in detail in our previous work [37,40]. The doping concentrations in this work all refer to the mass percentage.

## 4. Results and Discussion

**Figure 2:**
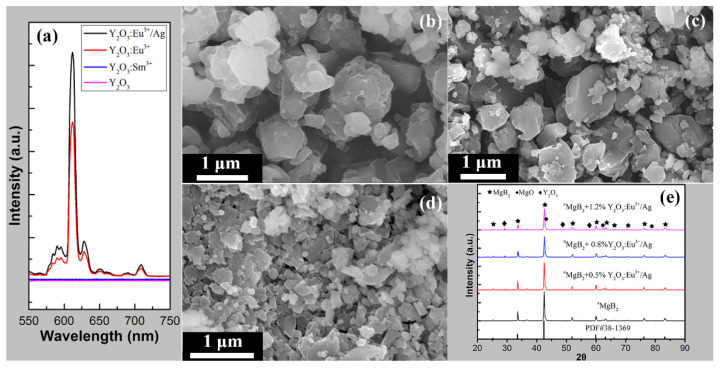
(**a**) EL intensities of Y_2_O_3_, Y_2_O_3_:Sm^3+^, Y_2_O_3_:Eu^3+^, and Y_2_O_3_:Eu^3+^/Ag. (**b**–**d**) SEM images of ^a^MgB_2_, ^b^MgB_2_, and ^c^MgB_2_. (**e**) XRD patterns of ^a^MgB_2_, ^a^MgB_2_ + 0.5% Y_2_O_3_:Eu^3+^/Ag, ^b^MgB_2_ + 0.8% Y_2_O_3_:Eu^3+^/Ag, and ^c^MgB_2_ + 1.2% Y_2_O_3_:Eu^3+^/Ag.

Figure 2a shows the EL spectra of Y_2_O_3_, Y_2_O_3_:Sm^3+^, Y_2_O_3_:Eu^3+^, and Y_2_O_3_:Eu^3+^/Ag, which confirm that Y_2_O_3_ and Y_2_O_3_:Sm^3+^ are non-EL materials, whereas Y_2_O_3_:Eu^3+^ and Y_2_O_3_:Eu^3+^/Ag show a remarkable EL property. Among the four materials tested, Y_2_O_3_:Eu^3+^/Ag showed the highest EL intensity because of the composite luminescence [44]. Figure 2b–d present the SEM images of the pure MgB_2_ samples prepared using three different raw materials. Figure 2b is the SEM image of ^a^MgB_2_, which shows that most of the particle exceeded 1 μm. For ^b^MgB_2_, only a few of the particles exceeded 1 μm as shown in Figure 2c. Figure 2d presents the SEM image of ^c^MgB_2_, which shows that most of particles are below 500 nm. The particle sizes of ^a^MgB_2_, ^b^MgB_2_, and ^c^MgB_2_ decrease in order. Figure 2e reveals the XRD patterns of four samples. The black and red curves depict the XRD patterns of ^a^MgB_2_ and ^a^MgB_2_ + 0.5% Y_2_O_3_:Eu^3+^/Ag, respectively. The blue and magenta curves correspond to the XRD patterns of ^b^MgB_2_ + 0.8% Y_2_O_3_:Eu^3+^/Ag and ^c^MgB_2_ + 1.2% Y_2_O_3_:Eu^3+^/Ag, respectively. The black vertical lines represent the standard XRD patterns of MgB_2_. The main phase of all the samples was clearly MgB_2_. The Y_2_O_3_ phase was found in the doped samples. Small amounts of the unavoidable MgO phase were also detected in all the samples [46,47,48,49]. The XRD patterns of the other samples show a similar feature.

**Figure 3:**
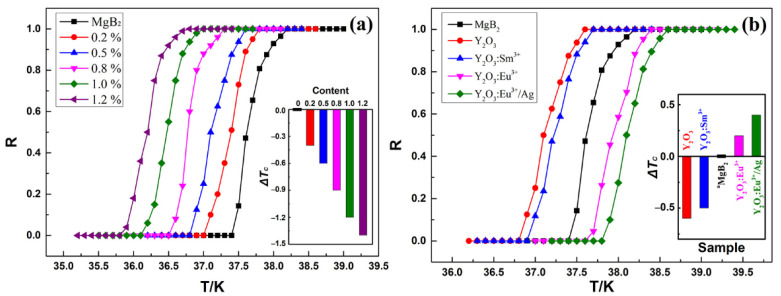
Normalized resistivity-temperature curves of ^a^MgB_2_ doped with (**a**) *x*% Y_2_O_3_ (*x* = 0, 0.2, 0.5, 0.8, 1.0, 1.2) and (**b**) 0.5% *y* (*y* = 0, Y_2_O_3_, Y_2_O_3_:Sm^3+^, Y_2_O_3_:Eu^3+^, Y_2_O_3_:Eu^3+^/Ag). Insets: the values of *ΔT_c_* (*ΔT_c_* = *T_c_* − *T_cpure_*).

Figure 3a illustrates the normalized resistivity-temperature (*R*–*T*) curves of ^a^MgB_2_ doped with *x*% Y_2_O_3_ (*x* = 0, 0.2, 0.5, 0.8, 1.0, 1.2). The black curve corresponds to the ^a^MgB_2_ sample, which shows that the *T_c_* of the pure sample was 37.4–38.2 K. The other curves represent ^a^MgB_2_ doped with Y_2_O_3_ with concentrations of 0.2%, 0.5%, 0.8%, 1.0%, and 1.2%, indicating that the corresponding *T_cs_* are 37.0–37.8 K, 36.8–37.6 K, 36.5–37.3 K, 36.1–37.0 K, and 35.8–36.8 K. The results show that like conventional chemical doping, the introduction of non-EL Y_2_O_3_ decreases the *T_c_* of MgB_2_ (*ΔT_c_* < 0) and tends to increase the superconducting transition width [50]. Meanwhile, the *T_cs_* of the doped samples decrease with the increase of the doping concentration as shown in the inset figure. Figure 3b shows the normalized *R*–*T* curves of ^a^MgB_2_ doped with 0.5% *y* (*y* = 0, Y_2_O_3_, Y_2_O_3_:Sm^3+^, Y_2_O_3_:Eu^3+^, Y_2_O_3_:Eu^3+^/Ag). The doping concentration was fixed at 0.5% base on our previous work [40]. The *T_c_* values of MgB_2_ doped with Y_2_O_3_, Y_2_O_3_:Sm^3+^, Y_2_O_3_:Eu^3+^, and Y_2_O_3_:Eu^3+^/Ag were 36.8–37.6 K, 36.9–37.7 K, 37.6–38.4 K, and 37.8–38.6 K. The results clearly show that non-EL Y_2_O_3_ and Y_2_O_3_:Sm^3+^ decreased the *T_c_* of MgB_2_, while EL Y_2_O_3_:Eu^3+^ and Y_2_O_3_:Eu^3+^/Ag increased the *T_c_* of MgB_2_, as shown in the inset. The *T_c_* values of MgB_2_ doped with Y_2_O_3_:Eu^3+^ and Y_2_O_3_:Eu^3+^/Ag increased by 0.2 and 0.4 K, respectively, compared with that of ^a^MgB_2_. This finding is similar to those of our previous studies.

**Figure 4:**
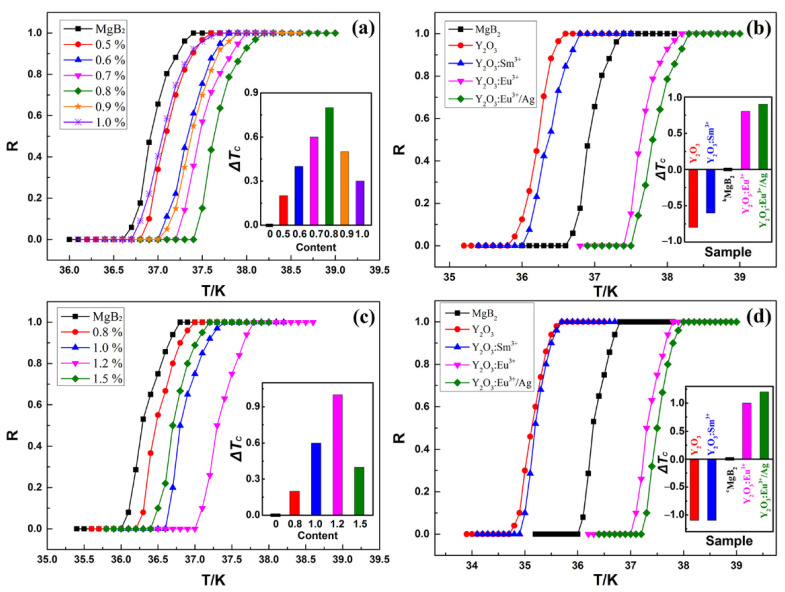
Normalized *R–T* curves of ^b^MgB_2_ doped with (**a**) *x*% Y_2_O_3_:Eu^3+^ (*x* = 0, 0.5, 0.6, 0.7, 0.8, 0.9, 1.0) and (**b**) 0.8% *y* (*y* = 0, Y_2_O_3_, Y_2_O_3_:Sm^3+^, Y_2_O_3_:Eu^3+^, Y_2_O_3_:Eu^3+^/Ag). Normalized *R–T* curves of ^c^MgB_2_ doped with (**c**) *x*% Y_2_O_3_:Eu^3+^ (*x* = 0, 0.8, 1.0, 1.2, 1.5) and (**d**) 1.2% *y* (*y* = 0, Y_2_O_3_, Y_2_O_3_:Sm^3+^, Y_2_O_3_:Eu^3+^, Y_2_O_3_:Eu^3+^/Ag). Insets: the values of *ΔT_c_* (*ΔT_c_* = *T_c_* − *T_cpure_*).

Figure 4a illustrates the normalized *R*–*T* curves of ^b^MgB_2_ doped with *x*% Y_2_O_3_:Eu^3+^ (*x* = 0, 0.5, 0.6, 0.7, 0.8, 1.0). The black curve corresponds to ^b^MgB_2_, which shows that the *T_c_* of the pure sample is 36.6–37.4 K. The other curves are the *R*–*T* curves of ^b^MgB_2_ doped with Y_2_O_3_:Eu^3+^ with doping concentrations of 0.5%, 0.6%, 0.7%, 0.8%, 0.9%, and 1.0%, indicating that the corresponding *T_cs_* are 36.8–37.6 K, 37–37.8 K, 37.2–38.0 K, 37.4–38.2 K, 37.0–37.9 K, and 36.7–37.7 K. The *T_c_* of the doped samples first increased and then decreased with the increase of the doping concentration. The inset summarizes the evolution of *ΔT_c_* as a function of the doping concentration. The optimal doping concentration and the corresponding *ΔT_c_* increased to 0.8% and 0.8 K, respectively, compared with those of the samples prepared using ^a^MgB_2_ as raw material. Figure 4b demonstrates the normalized *R*–*T* curves of ^b^MgB_2_ doped with 0.8% *y* (*y* = 0, Y_2_O_3_, Y_2_O_3_:Sm^3+^, Y_2_O_3_:Eu^3+^, Y_2_O_3_:Eu^3+^/Ag). The *T_cs_* of ^b^MgB_2_ doped with Y_2_O_3_, Y_2_O_3_:Sm^3+^, Y_2_O_3_:Eu^3+^, and Y_2_O_3_:Eu^3+^/Ag were 35.8–36.6 K, 36.0–36.8 K, 37.4–38.2 K, and 37.5–38.3 K, respectively. Among these samples, ^b^MgB_2_ + 0.8% Y_2_O_3_:Eu^3+^/Ag obtained the highest *ΔT_c_* (0.9 K) because of the high EL intensity, as shown in Figure 2a.

Figure 4c reveals the normalized *R*–*T* curves of ^c^MgB_2_ doped with *x*% Y_2_O_3_:Eu^3+^ (*x* = 0, 0.8, 1.0, 1.2, 1.5). Similarly, the black curve corresponds to the pure sample, indicating that the *T_c_* of ^c^MgB_2_ is 36.0–36.8 K. The other curves correspond to ^c^MgB_2_ doped with Y_2_O_3_:Eu^3+^ at different concentrations of 0.8%, 1.0%, 1.2%, and 1.5%, indicating that the corresponding *T_cs_* are 36.2–37.0 K, 36.6–37.4 K, 37.0–37.8 K, and 36.4–37.2 K, respectively. It is same with the results in Figure 3a, that is, *T_c_* first increases and then decreases with the increase of the doping concentration, as shown in the inset figure. The optimal doping concentration is 1.2%, and the corresponding *ΔT_c_* is 1.0 K. Figure 4d shows the normalized *R*–*T* curves of ^c^MgB_2_ doped with 1.2% *y* (*y* = 0, Y_2_O_3_, Y_2_O_3_:Sm^3+^, Y_2_O_3_:Eu^3+^, Y_2_O_3_:Eu^3+^/Ag). The *T_c_* values of ^c^MgB_2_ doped with Y_2_O_3_, Y_2_O_3_:Sm^3+^, Y_2_O_3_:Eu^3+^, Y_2_O_3_:Eu^3+^/Ag are 34.7–35.7 K, 34.9–35.7 K, 37.0–37.8 K, and 37.2–38.0 K. Y_2_O_3_ and Y_2_O_3_:Sm^3+^ decrease *T_c_*, whereas Y_2_O_3_:Eu^3+^ and Y_2_O_3_:Eu^3+^/Ag increase *T_c_*. These results are consistent with those of the samples prepared using ^a^MgB_2_ and ^b^MgB_2_ as raw materials. The *T_c_* of ^c^MgB_2_ + 1.2% Y_2_O_3_:Eu^3+^/Ag was enhanced by 1.2 K compared with that of the pure sample, exhibiting the highest *ΔT_c_* among the samples.

**Figure 5:**
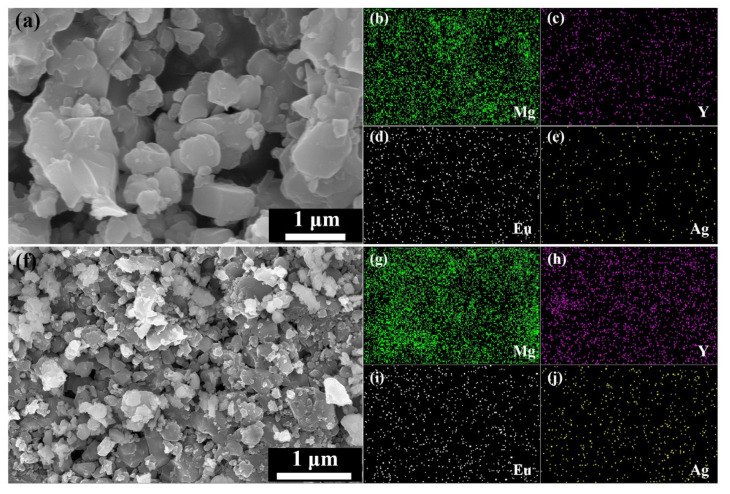
(**a**) SEM image and (**b**–**e**) EDS mapping of ^a^MgB_2_ + 0.5% Y_2_O_3_:Eu^3+^/Ag. (**f**) SEM image and (**g**–**j**) EDS mapping of ^c^MgB_2_ + 1.2% Y_2_O_3_:Eu^3+^/Ag.

Figure 5a shows the SEM image of ^a^MgB_2_ + 0.5% Y_2_O_3_:Eu^3+^/Ag. Figure 5b–e are the EDS mapping for elements Mg, Y, Eu, and Ag listed in the lower right corner of each figure. Figure 5h shows the SEM image of ^c^MgB_2_ + 1.2% Y_2_O_3_:Eu^3+^/Ag. Figure 5g–j are the EDS mapping for elements Mg, Y, Eu, and Ag. Given that the inhomogeneous phase did not react with MgB_2_, the mapping of elements Y, Eu, and Ag can reflect the distribution of the inhomogeneous phase in the sample. It can be seen that Y_2_O_3_:Eu^3+^/Ag is relatively evenly distributed in ^a^MgB_2_. Similarly, the inhomogeneous phase did not generate significant agglomeration in ^c^MgB_2_, even though the optimal concentration was enhanced to 1.2% as the particle size decreased, as shown in Figure 5g–j. Therefore, the inhomogeneous phase was able to fully exert the EL exciting effect to further increase *ΔT_c_* at high concentrations.

Table 1 shows the *ΔT_cs_* for ^a^MgB_2_ + 0.5% *x*, ^b^MgB_2_ + 0.8% *x*, and ^c^MgB_2_ + 1.2% *x* (*x* = Y_2_O_3_, Y_2_O_3_:Sm^3+^, Y_2_O_3_:Eu^3+^, and Y_2_O_3_:Eu^3+^/Ag). For the three kinds of MgB_2_ raw materials, non-EL dopants Y_2_O_3_ and Y_2_O_3_:Sm^3+^ can only decrease *T_c_* (*ΔT_c_* < 0) and the higher the doping concentration, the lower the *T_c_*. However, EL inhomogeneous phases can increase the *T_c_* (*ΔT_c_* > 0). For the ^a^MgB_2_ raw material, we prepared the MgB_2_ SMSCs doped with 0.5% inhomogeneous phase. The results show that *ΔT_c_* values for ^a^MgB_2_ doped with Y_2_O_3_:Eu^3+^ and Y_2_O_3_:Eu^3+^/Ag are 0.2 K and 0.4 K. For the ^b^MgB_2_ raw material with a smaller particle size than that of ^a^MgB_2_, the optimal doping concentration was first explored by changing the concentration of Y_2_O_3_:Eu^3+^ from 0.5% to 1.0%. The results show that the optimal doping concentration is 0.8%. Subsequently, 0.8% Y_2_O_3_:Eu^3+^, and Y_2_O_3_:Eu^3+^/Ag were separately doped into ^b^MgB_2_ and the corresponding *ΔT_c_* values were 0.8 K and 0.9 K, respectively. Similar results were obtained in the samples prepared using ^c^MgB_2_ as the raw material. For ^c^MgB_2_, which has the smallest particle size among the three raw materials, the optimal concentration was enhanced to 1.2%. The *ΔT_cs_* for ^c^MgB_2_ doped with Y_2_O_3_:Eu^3+^ and Y_2_O_3_:Eu^3+^/Ag were 1.0 K and 1.2 K, respectively. These results indicate that reducing the particle size can effectively increase the optimal doping concentration of the inhomogeneous phase, thereby enhancing the *ΔT_c_*.

In this work, the *ΔT_c_* is improved by increasing the optimal doping concentration of inhomogeneous phases through reducing the particle size, however, the *T_c_* values of MgB_2_ SMSCs are relatively low due to the low *T_c_* of the pure MgB_2_ sample. As the particle size decreases, the grain boundaries in the sample increase and the connectivity decreases, which are disadvantages to the superconductivity [51,52,53]. One possible solution is to incorporate the inhomogeneous phase into the interior of the particles to overcome the disadvantages caused by the increasing grain boundaries with the doping concentration increasing.

## 5. Conclusions

Although the effectiveness of improving the *T_c_* of superconducting materials through the SMSC method by doping with EL inhomogeneous phases has been proven in previous works, the *ΔT_cs_* obtained are quite small. To further increase *ΔT_c_*, three types of MgB_2_ raw materials, namely, ^a^MgB_2_, ^b^MgB_2_, and ^c^MgB_2_, were prepared with particle sizes decreasing in order. EL inhomogeneous phases were incorporated into these three raw materials with different concentrations to study the change of *ΔT_c_*. The results show that the optimal doping concentrations for ^a^MgB_2_, ^b^MgB_2_, and ^c^MgB_2_ are 0.5%, 0.8%, and 1.2%, respectively. The corresponding *ΔT_cs_* are 0.4, 0.9, and 1.2 K, respectively. Meanwhile, increasing the EL intensity of the inhomogeneous phase can be considered to further increase *ΔT_c_*. This work not only proves the effectiveness of the SMSC method in improving *T_c_* but also provides an alternative approach to improving the *T_c_* of superconducting materials.

## Figures and Tables

**Table 1:** *ΔT_cs_* for ^a^MgB_2_ + 0.5% *x*, ^b^MgB_2_ + 0.8% *x* and ^c^MgB_2_ + 1.2% *x* (*x* = Y_2_O_3_, Y_2_O_3_:Sm^3+^, Y_2_O_3_:Eu^3+^, and Y_2_O_3_:Eu^3+^/Ag).

ΔT_cs_	Y_2_O_3_	Y_2_O_3_:Sm^3+^	Y_2_O_3_:Eu^3+^	Y_2_O_3_:Eu^3+^/Ag
^a^MgB_2_ (0.5%)	−0.6 K	−0.5 K	0.2 K	0.4 K
^b^MgB_2_ (0.8%)	−0.8 K	−0.6 K	0.8 K	0.9 K
^c^MgB_2_ (1.2%)	−1.1 K	−1.1 K	1.0 K	1.2 K

## Data Availability

The data presented in this study are available on request from the corresponding author.
